# Postpartum Eclampsia Unresponsive to Magnesium Sulfate Therapy: An Uncommon Case Report

**DOI:** 10.7759/cureus.60047

**Published:** 2024-05-10

**Authors:** Sai Kalmegh, Meenal Patvekar

**Affiliations:** 1 Obstetrics and Gynecology, Dr. D Y Patil Medical College, Hospital and Research Center, Pune, IND

**Keywords:** resistance, eclampsia, seizure, postpartum eclampsia, magnesium sulfate

## Abstract

Despite advances in obstetric care, postpartum eclampsia continues to be a major cause of morbidity and mortality among mothers. Although the most common treatment for eclampsia during the postpartum phase is magnesium sulfate (MgSO4), a small percentage of individuals show resistance to this approach. Clinical challenges arise from MgSO4 resistance, which calls for different approaches to care to avoid unfavorable consequences for mothers. In this case study, we provide a thorough clinical description of a 19-year-old primigravida who gave birth at 37 weeks and developed postpartum eclampsia and did not improve with MgSO4 treatment. This case thus highlights the significance of accurate clinical diagnosis of patients and prompt use of alternative therapy modalities. We also discuss possible approaches to treating this uncommon but serious illness.

## Introduction

Preeclampsia is a condition specific to pregnancy and puerperium with blood pressure greater than 140/90 mmHg on at least two occasions four hours apart associated with proteinuria (24-hour urine protein excretion of more than 300 g) [1]. Eclampsia is a convulsive manifestation of preeclampsia that is associated with significant maternal morbidity and mortality in the intrapartum, antepartum, or postpartum period. Postpartum eclampsia remains a significant cause of maternal morbidity and mortality despite advancements in obstetric care. India has a staggering incidence of eclampsia, at 0.179%-3.7% and maternal mortality varies from 2.2% to 23% [2].

Although magnesium sulfate (MgSO4) is the standard treatment for managing eclamptic seizures in the postpartum period, there exists a subset of cases where patients demonstrate resistance to this therapy. MgSO4 resistance poses a clinical challenge because it necessitates alternative management strategies to prevent adverse maternal outcomes. Understanding the underlying mechanisms and identifying effective interventions for MgSO4-resistant postpartum eclampsia is imperative for improving maternal health outcomes. 

In this case report, we present a detailed clinical profile of a patient with MgSO4-resistant postpartum eclampsia, highlighting the challenges encountered in management and discussing potential strategies for addressing this rare but critical condition.

## Case presentation

A 19-year-old primigravida at 37 weeks of gestation was admitted to our labor room with complaints of headache and vomiting in the last 24 hours. The patient was referred from a primary healthcare center, where she was diagnosed with a case of gestational hypertension. Ten days prior to admission, the patient was on a tablet labetalol, 100 mg, once a day, although she had stopped two days before admission. A pulse rate of 72 beats per minute and blood pressure of 150/100 mmHg was recorded and bilateral pedal edema was noted. On per abdominal examination, she had a full-term uterus; the fetus was cephalic with a heart rate of 150 beats per minute. Vaginal examination revealed that the cervix was dilated 2-3 cm with minimal effacement and bulging membranes. The station was at -2 and the pelvis was adequate. Laboratory investigations revealed urine proteins +1, a urine protein creatinine ratio of 7.9, elevated PT/INR of 9.20/0.73, lactate dehydrogenase of 583 units/L, serum uric acid 8.3 mg/dl, a raised fibrinogen of 388 mg/dL, and a D-dimer of 1240 ng/mL. A diagnosis of impending eclampsia was made and a total loading dose of 14 g of MgSO4 was given. We followed up with a maintenance dose of 5 g four hours later. Induction with 0.5 mg of dinoprostone gel was carried out because she was term gestation with impending eclampsia. We conducted an emergency cesarean section for fetal tachycardia. The cesarean section was uneventful, and a 2.5 kg baby was safely delivered. In the immediate postoperative period, the patient had a generalized tonic-clonic seizure. Supportive therapy, that is, 4 g of MgSO4 at 20% solution was given intravenously over 15 minutes, following which the patient was stabilized and shifted immediately to the intensive care unit, where she had another tonic-clonic convulsion one hour after the first convulsion. Because the patient had not responded to MgSO4 therapy, we administered via intravenous route, 1 g injectable Levetiracetam and 10 mg Midazolam in addition to 20 mg injectable labetalol infusion to control blood pressure. The patient’s remaining postoperative period was uneventful, and she was discharged on day 10 on 500 mg tablet of Levetiracetam and 100 mg tablet of labetalol while maintaining a blood pressure of 120/80 mmHg.

## Discussion

Postpartum eclampsia is defined as the occurrence of convulsion in a woman with preexisting preeclampsia in the postpartum period [3]. MgSO4 has been the primary drug of choice for the treatment of eclampsia since the turn of the 20th century. It has multiple mechanisms of action including blocking ligand-gated calcium channels and N-methyl D-aspartate (NMDA) receptors and facilitating gamma amino butyric acid (GABA) receptors, thereby exerting an anticonvulsant effect. Clinical investigations have shown that MgSO4 is more effective than conventional anticonvulsant medications, such as phenytoin and diazepam, in treating and preventing eclamptic seizures. Recent controlled clinical trials and empirical evidence both support the efficacy of MgSO4 in treating eclamptic seizures [4]. MgSO4 has decreased the probability of recurrent seizures in eclamptic women by 52% compared to diazepam and by 67% compared to phenytoin in the Global Collaborative Eclampsia Trial [4].

According to Aya et al., eclampsia must be considered the default diagnosis in pregnant or postpartum women with seizures until proven otherwise. Cerebral venous thrombosis, reversible cerebral vasoconstriction syndrome, amniotic fluid embolism, thrombotic thrombocytopenic purpura, and air embolism are additional causes of pregnancy-related new-onset seizures [5].

In our case, the patient was a young primigravida at 37 weeks of gestation with impending eclampsia. In spite of receiving a loading and maintenance dose, the patient in the postpartum period had two seizure episodes. The patient was unresponsive to MgSO4 and hence we gave 1 g injectable Levetiracetam and 10 mg Midazolam intravenously to control the convulsions. We conducted an MRI of the brain to rule out other organic causes of the seizures, such as space-occupying lesions. In our patient, the MRI was within normal limits with no evidence of any pathological abnormality. The outcome, in this case, indicates that some women may have an inadequate response to MgSO4 and thus might require second-line drugs for control of convulsions.

A similar case series was reported by Alfonsus et al., wherein two patients had MgSO4-resistant eclampsia and were described to have required alternative antiepileptics, thiopental sodium, and Midazolam [6]. Similar to our case, the patient had an eclamptic attack postpartum after receiving the loading and maintenance dose of MgSO4. Midazolam was stopped on day 3, unlike our case where we continued Levetiracetam and Midazolam until day 5. In terms of current treatment guidelines, there is no stipulated protocol for management specific to eclampsia in the postpartum period when patients are unresponsive to MgSO4 therapy.

In women unresponsive to MgSO4, 1 g of Levetiracetam an anti-epileptic drug is used. Alternative drugs used in such women, as mentioned in Table 1, particularly with refractory eclampsia are thiopental sodium, an ultra-short-acting barbiturate, in a loading dose of 75-125 mg intravenously, and Midazolam, a benzodiazepine, in a loading dose of 10 mg intravenously [5].

**Table 1:** Other drugs used in management of eclampsia unresponsive to MgSO4. The table has been originally made by the authors with reference [6] IV: intravenous, h: hour, NMDA: N-methyl D-aspartate, GABA: Gamma Amino Butyric Acid, CNS: central nervous system, a2: alpha-2

Drug	Mechanism of action	Dose
Benzodiazepine (Diazepam, Midazolam)	Powerful anxiolytic, amnestic, hypnotic, anticonvulsant, skeletal muscle relaxant, and sedative properties	Loading dose: 10 mg IV over 2 min • Repeated if convulsion recurred • Maintenance dose: 40 mg in 500 mL normal saline for 24h Rate of infusion is titrated 20 mg in 500 mL for the next 24h and slowly reduced
		Midazolam Loading dose 0.2 mg/kg IV Maintenance dose 0.1 mg/kg/h IV
Barbiturate (Sodium Thiopental)	GABA agonist with possible actions on calcium channels NMDA receptor antagonist	*Loading dose: 75-125 mg IV bolus *Maintenance dose: 1-5 mg/kg/h IV 5 a2 agonist (Dexmedetomidine) * Loading dose: 1 mcg/kg per 20 min * Maintenance dose 0.7mcg/kg/h *400 mcg dexmetomidine is put in 100 ml normal saline
Propofol	Non-barbiturate anesthetic agent with anticonvulsant properties through potentiation GABA-mediated pre and post synaptic inhibition NMDA receptor antagonist	*Loading dose: 3-5 mg/kg IV * Maintenance dose 1- 15 mg/kg/h IV
a2 agonist (Dexmedetomidine)	Centrally-acting a2 agonist Sedative through locus coreolus in CNS	*Loading dose: 1 mcg/kg per 20 min *Maintenance dose 0.7 mcg/kg/h * 400 mcg dexmetomidine is put in 100 ml normal saline
Phenytoin	Stabilizing effect on neuronal membranes Recommended for prevention of convulsions in conjunction with 10 mg of diazepam for seizure attack	Loading dose of 15 mg/kg IV: 10 mg/kg initially in 30 minutes 5 mg/kg 2 hours later over 10 minutes Maintenance dose of 300 mg IV over 10 minutes until 24 hours post seizure Given in 70-100 ml of normal saline at rate of 25 mg/min

## Conclusions

Our case indicates that there are women with eclampsia who do not respond to MgSO4. In patients unresponsive to MgSO4, there are other drugs to prevent seizures. These cases highlight the importance of using drugs other than MgSO4 in the prevention and management of eclampsia. Our case thereby sheds light on alternative treatment options through scientific research and evidence-based medicine.
